# Effects of Propylene Glycol Alginate and Sucrose Esters on the Physicochemical Properties of Modified Starch-Stabilized Beverage Emulsions

**DOI:** 10.3390/molecules19068691

**Published:** 2014-06-24

**Authors:** Kok Whye Cheong, Hamed Mirhosseini, Nazimah Sheikh Abdul Hamid, Azizah Osman, Mahiran Basri, Chin Ping Tan

**Affiliations:** 1 Department of Food Technology, Faculty of Food Science and Technology, Universiti Putra Malaysia, UPM Serdang 43400, Selangor, Malaysia; E-Mail: hamedmi@upm.edu.my; 2 Department of Pharmaceutical Chemistry, School of Pharmacy, International Medical University, No. 126, Jalan Jalil Perkasa 19, Bukit Jalil, Kuala Lumpur 57000, Malaysia; E-Mail: CheongKokWhye@imu.edu.my; 3 School of Applied Sciences, AUT University, 34 St. Paul Street, Auckland 1142, New Zealand; E-Mail: nazimah.hamid@aut.ac.nz; 4 Department of Food Science, Faculty of Food Science and Technology, Universiti Putra Malaysia, UPM Serdang 43400, Selangor, Malaysia; E-Mail: azosman@upm.edu.my; 5 Department of Chemistry, Faculty of Science, Universiti Putra Malaysia, UPM Serdang 43400, Selangor, Malaysia; E-Mail: mahiran@upm.edu.my

**Keywords:** soursop beverage emulsion, propylene glycol alginate, sucrose esters, emulsion components, physicochemical properties

## Abstract

This study was conducted to investigate the effect of main emulsion components namely, modified starch, propylene glycol alginate (PGA), sucrose laurate and sucrose stearate on creaming index, cloudiness, average droplet size and conductivity of soursop beverage emulsions. Generally, the use of different emulsifiers or a mixture of emulsifiers has a significant (*p <* 0.05) effect on the response variables studied. The addition of PGA had a significant (*p <* 0.05) effect on the creaming index at 55 °C, while PGA-stabilized (PGA1) emulsions showed low creaming stability at both 25 °C and 55 °C. Conversely, the utilization of PGA either as a mixture or sole emulsifier, showed significantly (*p <* 0.05) higher cloudiness, as larger average droplet size will affect the refractive index of the oil and aqueous phases. Additionally, the cloudiness was directly proportional to the mean droplet size of the dispersed phase. The inclusion of PGA into the formulation could have disrupted the properties of the interfacial film, thus resulting in larger droplet size. While unadsorbed ionized PGA could have contributed to higher conductivity of emulsions prepared at low pH. Generally, emulsions prepared using sucrose monoesters or as a mixture with modified starch emulsions have significantly (*p* < 0.05) lower creaming index and conductivity values, but higher cloudiness and average droplet size.

## 1. Introduction

Soursop (*Annona muricata*) is a tropical fruit native to and common in tropical America and the West Indies, although it is grown in several other countries, including Sri Lanka [1]. Today, it is widely distributed, from southeastern China to Australia and the warm lowlands of eastern and western Africa and Southeast Asia. Of the 60 or more species of the genus *Annona*, family Annonaceae, only five species, namely soursop, custard apple, cherimoya, bullock’s heart and atemoya are of commercial importance and have contributed to significant economic growth of certain tropical countries, namely tropical America, northern South America, Australia, Africa and Malaysia [2,3]. Nevertheless, only soursop, which is the largest-fruited species, lends itself well to preserving and processing.

Generally, soursop has a pleasant, sub-acid and aromatic flesh that is described as a custard-like flavor when it is ripe, due to the presence of ester compounds, fruit sugars, organic acids and uncharacterized volatile esters [4]. The soursop fruit has been widely used for manufacturing various types of products such as juice blends, puree, jellies, syrups, jams and ice-cream [5]. Like most tropical fruits, the unique soursop flavor implies that the fruit itself or its processed products would have great potential to compete in the international market [6]. Soursop pulp has already been marketed in European, North American and Brazilian markets [7]. In year 2009, the food and beverages was accounted for nearly US$ 9 billion of the total flavor and fragrance sales. Considering the fact that Malaysia has an abundance of tropical fruits with distinctive flavors and the advancement of food and beverage processing, the food and beverages sector will continue to represent the largest market share in utilizing flavoring agents [8].

An emulsion is a suspension of two immiscible liquids (water and oil) in which small oil droplets is dispersed in an aqueous phase or vice versa [9]. Owing to the relatively simple formation and processing methods, colloidal delivery system through emulsion has been widely utilized as an efficient delivery method in the food and beverage industries. Amongst the various food ingredients, flavorings have been one of the most important and valuable ingredients in any food products. As flavor compounds can be expensive and delicate, protection of these valuable ingredients from evaporation, degradation and oxidation has often been a cause of concern for manufacturers [10,11]. Besides that, emulsion system allowed the dispersion of oil-based flavor compounds into aqueous food systems (e.g., soft drinks, beverage emulsion).

Beverage emulsions are oil-in-water (o/w) emulsions that are normally prepared in concentrated form and then diluted several hundred times prior to the consumption either in carbonated or non-carbonated soft drinks. In the soft drinks industry, the concentrated emulsion is diluted several hundred to several thousand times in a sugar/acid solution to provide flavor, color and a cloudy appearance to the final products [12]. The beverage emulsions must have a high degree of stability in both concentrated and diluted forms [10]. However, emulsions are thermodynamically unstable systems due to unfavorable contact and the differences in specific gravity between the oil and aqueous phase, leading to separation, decrease of both aesthetic and flavor quality over a period of time [9]. As the physicochemical stability of emulsions is of vital importance to industry manufacturers, long term stability is often extended by improvement of their kinetic stability through addition of emulsifiers and/or thickening agents to retard droplet aggregation or flocculation and coalescence [9,13]. Beverage emulsions are often stabilized by conventional amphiphilic and anionic hydrocolloids such as gum arabic, xanthan gum and modified starch [14,15,16]. But in recent years, various types of biopolymers such as corn fiber gum [17], durian seed gum [18], fish gelatin [19], alginates [20] or the use of a mixture of polysaccharide-protein systems [21,22,23] have been proposed as alternative sources of natural emulsifiers. The global consumption of 412 billion liters of soft drinks in 2001 represented by any measure the majority of the beverage industry, which also reflects on the use of biopolymers in the industry [24].

Starches can be surface-active when modified with hydrophobic moieties such as octenylsuccinic anhydride (OSA) [25]. For food application purposes, the chemical modification of starch is strictly limited to the type of chemical reaction, modifying agent, degree of substitution and the impurity content [26], with a typical degree of substitution up to 0.02% or 3% [27,28]. Trubiano touted hydrophobically modified starches as one of the most promising biopolymer replacements for gum arabic, and it is perhaps the most widely accepted alternative for beverage emulsion applications [29]. With the incorporation of a lipophilic group, the OSA-modified starch shows surface-active properties that can be used to stabilize o/w emulsions [30,31]. Previous studies have indicated that modified starch is mildly anionic in aqueous solutions and has a surface activity that is almost as high as that of gum arabic [32]. Modified starch forms a strong film at the oil-water interface, which resists the re-agglomeration of newly formed emulsion droplets [27].

Alginates, hydrophilic colloidal polysaccharides that act as structural components, are found abundantly in various species of marine brown seaweeds (*Phaeophyceae*). Alginates are linear polysaccharides comprising (1-4)-linked β-d-mannuronate and α-l-guluronate binary copolymers at varying proportions and distributions in the chain [20]. However, the only alginate derivative that is highly in demand among beverage manufacturers is propylene glycol alginate (PGA) because sodium alginates do not function below pH 4.0. By esterifying the alginate and propylene oxide to form PGA, this derivative can function as an emulsifier at lower pH and is also highly soluble [33]. Other attractive properties of PGA include viscosity enhancement, stabilization and film formation [20,34]. Sucrose fatty acid esters, better known as sucrose esters, are non-ionic emulsifiers that have been widely applied in the food industry in recent years because they are biodegradable, of low toxicity, tasteless and odorless [35]. These emulsifiers can be produced from natural products by the esterification of fatty acids or natural glycerides with sucrose and are therefore perceived as more environmentally friendly [36,37]. As such, these surfactants can be synthesized with various hydrophobic-hydrophilic and functional properties using different types of fatty acids [37,38]. By varying the number of fatty acids from one to eight and the chain length of the fatty acids, sucrose esters with a wide range of hydrophilic-lipophilic balance (HLB) values and properties can be synthesized to accommodate applications in o/w or w/o emulsion systems [35,37]. Given the right composition and temperature, previous studies have also shown the versatility of sucrose monoesters in the formation of various types of colloidal dispersions such as microemulsions, nanoemulsions and emulsions [39].

The physicochemical properties of emulsions, such as beverage emulsions, are important because they dictate the quality of the products and their aesthetic value. The characteristics of emulsion products do not depend on the type and concentration of each individual component but rather on the combination of all the components together in an emulsion system and the interactions between these components [9]. The stability of beverage emulsions stabilized by a single emulsifier system is often limited. Thus, the development of more refined dispersion technologies which utilizes complexes or mixed biopolymers, as well as the incorporation of small molecules (e.g., sucrose esters and maltose) has become ever more important [19,23,34,40,41]. Though steric hindrance may play a key role in emulsion formed using OSA-modified starch [42], the stability of the emulsion is also related to the behavior of the continuous aqueous phase such as ionic environment, rheology and unadsorbed biopolymers or surfactants [43]. There may be synergistic or antagonistic interactions between different kinds of biopolymers that will cause large changes in their functional properties [44].

Taherian *et al*. reported that the addition of xanthan gum to the modified starch-stabilized emulsion had provided greater stability by increasing the elastic modulus (G’) of the emulsion. Besides that, xanthan gum-added emulsion also had smaller particle size. This is mainly attributed to the immobilization of the starch-coated oil droplets in a weak gel-like network formed by the xanthan gum [45]. Alimi *et al*. observed a highly monodisperse packed structure with larger average droplet size when the low-fat mayonnaise was prepared using inulin of longer chain length and higher concentration of both starch and inulin [46]. A previous report found that the addition of maltose could retard the modified starch-stabilized emulsion against creaming, flocculation and coalescence [41]. Maltose had increased the viscoelasticity of the continuous phase, hence retarded free motion of droplets. In addition, the interfacial characteristics were also reported to be dependent on the procedures and/or types of emulsifiers used to make emulsions [40,47]. While protein-polysaccharide [19,34,48] and protein-sucrose ester [40,49] interactions have been widely reported, interactions between modified starch and sucrose esters, as well as PGA remained unclear. In addition, there were issues in utilizing sucrose esters in acidic beverage emulsions, although Choi *et al*. recently reported a stable emulsion when sucrose palmitate was used in combination with lyso-lecithin [50]. The addition of anionic lyso-lecithin had significantly increased the electrostatic repulsion between droplets, which consequently improved the stability of the emulsion against droplet aggregation and phase separation at low pH. Thus, the main aim of the present study was to study the effect of emulsion compositions and the interactions between these components on the physicochemical properties of soursop beverage emulsion (e.g., creaming index, cloudiness, average droplet size and conductivity). In this study, a mixed emulsifier system consisting of modified starch with PGA, sucrose laurate or sucrose stearate were investigated. In addition, emulsions stabilized by 1% (w/w) of emulsifiers were also prepared as sucrose esters were known for their emulsification efficiencies at low concentration [50,51].

## 2. Results and Discussion

### 2.1. Creaming Index

Prior to the creaming index study, several variables (e.g., speed of the homogenizer, duration of the shearing and pressure of the high-pressure homogenizer) that could affect emulsion preparation were optimized. The conditions that led to the highest creaming stability, narrow polydispersity and minimum mean droplet size (average droplet size < 1 μm) were chosen as indicators. The results showed that a fine emulsification with a good creaming stability could be achieved by mixing the emulsion for 1 min using a high-shear homogenizer (6,000 rpm) before being sent through a high-pressure homogenizer for two cycles at 200 bar.

**Table 1:** The experimental values for the response variables (*Y_j_*) (mean ± SD).

Samples	Physicochemical properties					
	Creaming Index at 10 °C (%)	Creaming Index at 25 °C (%)	Creaming Index at 55 °C (%)		Cloudiness (Ǻ)	Conductivity (mS/cm)
MS6	100.0 ± 0.0 ^a^	100.0 ± 0.0 ^a^	86.0 ± 1.4 ^a^		0.091 ± 0.004 ^a^	0.0749 ± 0.0001 ^a,e^
MS-PGA	97.5 ± 3.5 ^a^	98.5 ± 2.1 ^a^	51.0 ± 1.4 ^b^		0.111 ± 0.001 ^a^	0.0837 ± 0.0002 ^b^
MS-SL	16.5 ± 2.1 ^b,d^	10.5 ± 0.7 ^b^	11.0 ± 1.4 ^c^		0.160 ± 0.013 ^b^	0.0719 ± 0.0000 ^c,d^
MS-SS	36.5 ± 2.1 ^c^	16.5 ± 2.1 ^c^	10.5 ± 0.7 ^c^		0.163 ± 0.001 ^b^	0.0742 ± 0.0000 ^a,d^
MS1	23.5 ± 2.1 ^b^	20.5 ± 0.7 ^c^	20.5 ± 0.7 ^d^		0.160 ± 0.004 ^b^	0.0725 ± 0.0006 ^d^
PGA1	99.5 ± 0.7 ^a^	41.5 ± 2.1 ^d^	14.5 ± 0.7 ^c^		0.309 ± 0.018 ^c^	0.0766 ± 0.0001 ^e^
SL1	14.0 ± 1.4 ^d^	10.5 ± 0.7 ^b^	10.5 ± 0.7 ^c^		0.089 ± 0.013 ^a^	0.0695 ± 0.0001 ^f^
SS1	36.0 ± 1.4 ^c^	31.0 ± 1.4 ^e^	21.5 ± 2.1 ^d^		0.110 ± 0.000 ^a^	0.0682 ± 0.0011 ^f^
**Samples**	**Physicochemical properties**					
	**Average Droplet Size, D[4,3] (µm)**			**Average Droplet Size, D[4,3] (µm)**		
MS6	0.245 ± 0.005 ^a^		0.299 ± 0.003 ^a^			
MS-PGA	0.448 ± 0.016 ^a^		0.849 ± 0.061 ^b^			
MS-SL	1.526 ± 0.004 ^b^		42.178 ± 0.130 ^c^			
MS-SS	5.992 ± 0.045 ^c^		35.250 ± 0.040 ^d^			
MS1	8.476 ± 0.186 ^d^		8.535 ± 0.056 ^e^			
PGA1	2.179 ± 0.014 ^e^		2.527 ± 0.101 ^f^			
SL1	8.356 ± 0.059 ^d^		21.014 ± 0.204 ^g^			
SS1	1.815 ± 0.021 ^f^		10.671 ± 0.221 ^h^			

Different lowercase letters (a–h) showed significant difference between variables of the same physicochemical properties by different emulsifiers or a combination of emulsifiers at *p* < 0.05.

The results showed that the use of different emulsifiers or a combination of emulsifiers has a significant (*p* < 0.05) effect on emulsion stability at different temperatures (Table 1). In general, there were no significant differences (*p* > 0.05) between formulations stabilized using 6% (w/w) modified starch (MS6) or a mixture of modified starch and propylene glycol alginate (PGA) (MS-PGA) at low or at room temperature. However, as the storage temperature increased to 55 °C, MS-PGA exhibited a significantly (*p* < 0.05) lower stability, although the viscosity of MS-PGA increased significantly (*p* < 0.05) compared with that of MS6 (data not shown). This result could be due to the depletion flocculation induced by the excess polysaccharides and the inability of PGA to form a three-dimensional gel network that would retard the movement of dispersed droplets at a higher temperature [52,53].

In the PGA-stabilized emulsion (PGA1), the creaming stabilities decreased as the temperature increased. The creaming stability of PGA1 at room temperature was 41.5%, in stark contrast with that of PGA at 95% after 3 months of storage at similar concentrations [54]. In addition, the creaming stability at 55 °C had also decreased significantly (*p* < 0.05) to 14.5%. This result concurred well with Paraskevopoulou *et al*., who had reported a lower interfacial activity of PGA in stabilizing olive oil droplets [55]. They found that gum arabic and egg yolk were more effective and performed as better emulsifiers than PGA. Propylene glycol alginate was unable to protect the dispersed droplets from coalescence as PGA molecules were unable to adsorb at the oil droplet surface to form a film of high surface shear viscosity [55]. The lower stability of MS1 (1% w/w modified starch) was mainly due to the insufficient amount of emulsifier present at the oil-water interface to stabilize the dispersed droplets.

On the other hand, emulsions prepared using sucrose esters (SL1 and SS1) or a mixture of sucrose esters and modified starch (MS-SL and MS-SS) were unstable in terms of creaming (Table 1). In general, there were no significant differences (*p* > 0.05) between emulsions prepared using MS-SL and SL1. However, there were significant differences (*p* < 0.05) in the creaming stability index between MS-SS and SS1 when stored at 25 °C and 55 °C. The higher instabilities at room and high temperatures could have been due to the higher effective collision frequency and efficiency between droplets as a result of their Brownian motion [9]. In addition, samples stabilized using only sucrose stearate (SS1) or mixtures of modified starch with sucrose stearate (MS-SS) were generally much more stable than their sucrose laurate counterparts. This result could be due to the higher solubility of sucrose stearate in the water phase, consequently of lower HLB value than sucrose laurate, which lead to a higher amount of emulsifier available to stabilize the dispersed droplets. This is in contrast with the observations made by Leong *et al*. [56], as emulsifiers with higher HLB values should be able to stabilize dispersed emulsion droplets more efficiently and rapidly [57].

The comparisons between MS6, MS-SL and MS-SS clearly demonstrated the antagonistic effect of sucrose esters in the formulation of a soursop beverage emulsion system. This finding can mainly be attributed to the instability of sucrose esters at low pH. Noritomi *et al*. observed that sucrose monoesters tend to be chemically unstable at low and high pHs [58]. They found that sucrose monoesters are susceptible to the hydrolysis of ester bonds at alkaline pHs and hydrolysis of the sucrose head group in acidic pH environments. As such, in an acidic environment, the size of the hydrophilic head group would be reduced, thus reducing the steric repulsion between dispersed droplets. Additionally, impurities present in sucrose esters such as lauric and stearic acid, which are expected to be surface-active, will remain predominantly in the neutral form (R-COOH) when the pH < pKa. Thus, the net charge would be close to zero, and electrostatic repulsion between the droplets would be rendered ineffective. Several reports suggest that the pKa values for lauric and stearic acids are 7.50 and 10.15, respectively [59,60]. Similar observations were made by Rao and McClements for a sucrose monopalmitate emulsion preparation [39].

### 2.2. Cloudiness

As shown in Table 1, the emulsions stabilized by MS6 or MS-PGA were significantly (*p* < 0.05) less cloudy than other samples. The results showed that the cloudiness was directly proportional to the mean droplet size of the dispersed phase, except for SL1 and SS1 (Table 1). This result is in agreement with previous reports that the type and concentration of hydrocolloids could consequently affect the average droplet size as well as the refractive index of the oil and aqueous phases and thus the cloudiness of the beverage emulsion [61]. However, SL1 and SS1, which were stabilized using sucrose laurate and stearate, respectively, were found to have a low cloudiness value, even though these emulsions were reported to have a larger mean droplet size than MS6 and MS-PGA (Table 1). This result was unexpected, as an emulsion containing a larger average droplet size and distribution would be expected to have a greater cloudiness value than the emulsion with a smaller mean droplet size.

On the other hand, the higher degree of cloudiness of MS-SL, MS-SS, MS1 and PGA1 was in good agreement with the generally larger mean droplet size. Thus, the results showed an antagonistic effect of sucrose monoesters and modified starch (MS-SL and MS-SS) in contributing to the cloudiness of soursop beverage emulsions. This finding could be explained by the competitive adsorption between modified starch and sucrose monoesters onto the oil-water interface, creating an irregular oil-water interface and therefore a larger mean droplet size. Similar observation was also reported by Zhao *et al*. [40]. They observed a significant (*p* < 0.05) change in the distribution of average droplet size and the specific surface area has increased significantly (*p <* 0.05) from 10.1 to 11.4 m^2^/g with increasing sucrose ester concentration [40]. Hence, the properties of the interfacial films are dependent on the compositions and interactions between surface active materials at the oil-water interface [56]. Additionally, the instability of sucrose monoesters in acidic conditions contributed to a larger mean droplet size and hence, the higher degree of cloudiness of MS-SL and MS-SS.

### 2.3. Average Droplet Size

Stabilized emulsions formed by MS6 and MS-PGA have the smallest (*p* < 0.05) average droplet size (Table 1). A comparison of MS6 and MS-PGA showed that the inclusion of PGA in the formulation could have disrupted the arrangement of the more readily adsorbed modified starch at the oil-water interface, resulting in a slightly larger particle size. The significantly (*p* < 0.05) larger average droplet size of MS1 was mainly due to the limiting amount of emulsifier present to stabilize the newly formed dispersed droplets, thus leading to a higher collision frequency and coalescence [9]. Table 1 shows a significantly (*p* < 0.05) larger mean droplet size for PGA1 compared with that of MS6 or MS-PGA. This observation could be explained by the fact that PGA has a relatively lower surface activity when compared to modified starch; thus, the PGA-stabilized emulsion exhibited a larger mean droplet size [55]. This is further confirmed by the significantly (*p* < 0.05) larger average droplet size of both MS-PGA and PGA1 after two months of storage.

The sucrose monoester-based emulsions (SL1 and SS1) clearly showed the ineffectiveness of sucrose esters as emulsifiers at low pH. After two months of storage, the average droplet size for both SL1 and SS1 had increased significantly (*p* < 0.05) (Table 1). The hydrolysis of the sucrose head group at low pH rendered the effectiveness of the steric repulsion mechanism, while the fatty acids remained predominantly a neutral molecule when pH < pKa of both lauric and stearic acids [58]. Besides that, more droplet flocculation in sucrose esters-stabilized emulsions was also suggested to be a reason of sucrose esters-stabilized emulsions having larger average droplet size [51]. However, the significantly smaller (*p* < 0.05) mean droplet size of SS1 could be attributed to the higher solubility of sucrose stearate in the water phase, thus, providing a larger amount of emulsifier to stabilize the newly formed droplets. Our observations were in complete contrast to reports by Leong *et al*., in which sucrose laurate was shown to be a better emulsifier compared with sucrose stearate, due to its higher particle entrapment and stabilization efficacies [56]. However, sucrose stearate had an antagonistic effect when used in combination with modified starch (MS-SS), as shown by a significantly (*p* < 0.05) larger average droplet size compared with that of MS-SL. This result could be due to the hydrophobic interaction between the amylopectin of modified starch and stearic acid at the oil-water interface. Extensive hydrophobic interactions at the oil-water interface could eventually lead to coagulation of modified starch and sucrose stearate, forming multilayers of modified starch and sucrose stearate. This eventually will reduce the surface active properties of both modified starch and sucrose stearate and easily be displaced [62]. In addition, the average droplet size is also influenced by the interfacial tension, which is mainly characterized by the compositions and properties of the interfacial films [56]. Similarly, Zhao *et al*. reported that the added sucrose esters could be co-adsorped or competitively adsorb onto the sodium caseinate emulsion interface, hence, changing the compositions and properties of the interfacial films, and consequently altering the average droplet size [40].

### 2.4. Conductivity

As shown in Table 1, both MS-PGA and PGA1 showed a significantly (*p* < 0.05) higher conductivity compared with that of the other samples. MS-PGA had the highest (*p* < 0.05) conductivity value among all of the samples. This result could be due to the adsorption of the more surface-active modified starch onto the oil-water interface, restricted further adsorption of PGA through steric hindrance caused by the highly branched amylopectin. In addition, turbulent flow factor caused by high shear homogenization and high pressure homogenization also favors the transport of more surface-active molecules, which lead to the higher surface load of modified starch at the emulsion interface [63]. Thus, the ionized PGA was retained mainly in the continuous phase and contributed to the higher conductivity of MS-PGA. Similar observation had been made by Mirhosseini *et al*., as they reported that the interaction between gum arabic and xanthan gum had exhibited the most significant (*p* < 0.05) effect on the conductivity [15].

Meanwhile, MS6, MS-SL, MS-SS and MS1, showed significantly (*p* < 0.05) lower conductivities due to the mildly charged modified starch, which introduced a lower charge into the system. On the other hand, sucrose laurate and stearate (SL1 and SS1) contributed the least (*p* < 0.05) to the conductivity of emulsion system. This result was expected because the fatty acids would remain mainly as neutral molecules (R-COOH) in acidic conditions when the pH < pKa [39,50,58].

## 3. Experimental

### 3.1. Materials

Octenylsuccinic anhydride (OSA) modified waxy maize starch (Purity Gum 1773) was a kind gift of National Starch and Chemical (Bridgewater, NJ, USA). Propylene glycol alginate (PGA, Protanal ester SD 8440) was provided by FMC Biopolymer (Philadelphia, PA, USA). Sucrose ester emulsifiers, sucrose laurate (L-1695) (HLB value = 16) with an 80% monoester content and sucrose stearate (S-1570) (HLB value = 15) with a 70% monoester content were gifts from Mitsubishi-Kagaku Foods Corporation (Tokyo, Japan). The soursop flavor oil was provided by Flavor Inn Corporation (Selangor, Malaysia). Vegetable oil (palm olein) was purchased from a local retailer. Citric acid (Sigma-Aldrich, St. Louis, MO, USA) was used to adjust the pH of the emulsions. Sodium benzoate (Sigma-Aldrich) and potassium sorbate (Acros Organics, Fair Lawn, NJ, USA) were used as preservatives in the beverage emulsion systems.

### 3.2. Preparations of Soursop Beverage Emulsions

Two soursop beverage emulsions composed of modified starch (6% w/w and 1% w/w), soursop flavor oil (10% w/w), vegetable oil (3% w/w), citric acid (0.4% w/w), sodium benzoate (0.1% w/w), potassium sorbate (0.1% w/w) and deionized water were prepared as control samples (Table 2). In other formulations, a 1% (w/w) emulsion of PGA, sucrose laurate and sucrose stearate was used in the sole emulsifier systems. In a mixed system, 6% (w/w) of modified starch was used in combination with 1% (w/w) of either PGA, sucrose laurate or sucrose stearate, along with other basic emulsion components.

**Table 2:** The matrix of experimental design.

Sample Name	Modified Starch (% w/w)	PGA (% w/w)	Sucrose Laurate (% w/w)	Sucrose Stearate (% w/w)	Soursop Oil (% w/w)	Vegetable Oil (% w/w)
MS6 *	6.00	−	−	−	10.00	3.00
MS-PGA	6.00	1.00	−	−	10.00	3.00
MS-SL	6.00	−	1.00	−	10.00	3.00
MS-SS	6.00	−	−	1.00	10.00	3.00
MS1 *	1.00	−	−	−	10.00	3.00
PGA1	−	1.00	−	−	10.00	3.00
SL1	−	−	1.00	−	10.00	3.00
SS1	−	−	−	1.00	10.00	3.00

* Control sample.

To prepare the aqueous phase, sodium benzoate, potassium sorbate and citric acid were sequentially dispersed in deionized water that was kept stirred using a magnetic stirrer. Subsequently, modified starch and/or PGA, sucrose laurate or sucrose stearate were also dispersed in succession in the deionized water. The mixture was then left at room temperature while being stirred for 2 h to facilitate hydration. While mixing the aqueous phase using a high speed Waring blender (32BL80, New Hartford, NY, USA), the soursop flavor oil was gradually added into the aqueous phase to form an initial coarse emulsion. Fine emulsification (e.g., small average droplet size of <1 µm with a narrow particle size distribution) was achieved by subjecting the initial coarse emulsion to pre-homogenization using a high-shear homogenizer (Silverson L4R, Buckinghamshire, UK) for 1 min at 6,000 rpm and then passing it through a high-pressure homogenizer (APV, Crawley, UK) for 2 cycles at 200 bar. The experimental design is given in Table 2, and all of the formulations were prepared in duplicate.

### 3.3. Creaming Index

The physical stability of soursop beverage emulsions was determined by measuring the extent of gravitational phase separation. The creaming and sedimentation values were determined from the ratio of cream and sediment volumes over the total volumes of emulsion samples [64]. An approximate 10 mL of emulsion samples were transferred into a 15-mL test tube and monitored for 2 months under refrigerated temperature (10 ± 3 °C), room temperature (25 ± 3 °C) and high temperature storage conditions (55 ± 3 °C). The emulsions were monitored by visual observation, and the stability index was calculated as a percentage of the initial emulsion height (HE), the height of the cream layer (HC) and the height of the sedimentation phase (HS) [54]:


Emulsion stability (%) = (remaining emulsion height/initial emulsion height) × 100
(1)

The higher emulsion stability against creaming was demonstrated by the larger emulsion stability value. The measurement was performed in triplicate. The average of measurements was used as the response value for data analysis.

### 3.4. Cloudiness

The concentrated soursop beverage emulsions were diluted to 0.1% (w/w) using deionized water prior to measurements using a UV-visible spectrophotometer at 660 nm (Spectronic Genesys™ 10, GENEQ Inc., Montreal, QC, Canada). The cloudiness was then calculated from the absorbance readings, as described by Garti *et al*. [65]. Higher absorbance readings correspond to a higher cloudiness in the beverage emulsions. The absorbance readings were also performed in triplicate.

### 3.5. Average Droplet Size

The average droplet size of the emulsions were measured using a Malvern Mastersizer 2000 particle size analyzer (Malvern Instrument Limited, Worcestershire, UK) equipped with a Hydro 2000 MU sample dispersion unit. This instrument measures the intensity of laser light scattered from dilute emulsion samples and reports the particle size distribution that gives the best fit between theoretical (Mie theory) and experimental values of intensity *versus* scattering angle [66]. To avoid multiple scattering effects, the measurements were conducted by adding the concentrated emulsions dropwise into the sample dispersion unit until an obscuration value of between 1% and 2% was reached. The average droplet size measurements were reported as volume-weighted means, D[4,3] or also known as the De Brouckere mean diameter. The measurements of the droplet size were performed immediately after preparation and were reported as the average of two separate measurements, with five readings made for each measurement.

### 3.6. Conductivity

The concentrated soursop emulsions were diluted (1% w/w) for the measurement of the conductivity using a Malvern Zetasizer Nano Z (Malvern Instruments Limited). The measurement unit for conductivity is mS/cm. The measurements were taken after the instrument was equilibrated for 1 min at 25 ± 1 °C. The measurements were performed immediately after the preparation of the emulsion. The experimental data for conductivity were reported as the average of two separate measurements, with five readings made for each measurement.

### 3.7. Statistical Analysis

The data obtained from the measurements were subjected to analysis of variance (ANOVA) to determine the significant differences among the samples. Each of the duplicated formulations will be subjected to triplicate measurements. Then the mean taken from each sample analysis were subjected for further ANOVA analysis and the results were reported as the mean ± standard deviation. Differences were considered significant if *p* < 0.05. The data analysis was performed using Minitab release 14.20 statistical package (Minitab Inc., State College, PA, USA).

## 4. Conclusions

The results showed a significant (*p* < 0.05) effect of PGA and sucrose esters on the physicochemical properties of soursop beverage emulsions, whether used as the sole emulsifier or in combination with modified starch. Emulsions stabilized solely by modified starch (MS6) showed good emulsion characteristics, with excellent creaming stability, cloudiness and average droplet size at all temperatures. The results also indicated that PGA may be used in combination with modified starch to modulate the physical stability of emulsion at low and room temperatures. However, PGA1 showed a significantly (*p* < 0.05) lower creaming stability at room and higher temperatures. These results indicated that PGA was not as surface-active as modified starch. Sucrose monoesters were found to be unsuitable for emulsion stabilization at low pH conditions, either in a mixture or as the sole emulsifier. Emulsions stabilized by a mixture of modified starch and sucrose esters showed a significantly (*p* < 0.05) higher average droplet size and lower creaming stability. The incorporation of sucrose esters had altered the emulsion interface, leading to destabilization of the emulsions. Sucrose ester-stabilized emulsions (SL1 and SS1) also exhibited significantly (*p* < 0.05) lower conductivity as the fatty acids would remain mainly as neutral molecules (R-COOH), which hinder stabilization through electrostatic repulsion mechanisms. Though the incorporation of PGA and sucrose esters had a negative impact on the physicochemical properties, this study showed the importance of understanding the interaction effects between emulsion components for developing an optimum beverage emulsion with desirable physicochemical properties.
